# Safety of ferric derisomaltose and iron sucrose in patients with iron deficiency anemia: The FERWON‐IDA/NEPHRO trials

**DOI:** 10.1002/ajh.26015

**Published:** 2020-10-27

**Authors:** Myles Wolf, Michael Auerbach, Philip A. Kalra, John Glaspy, Lars L. Thomsen, Sunil Bhandari

**Affiliations:** ^1^ Division of Nephrology Department of Medicine, and Duke Clinical Research Institute, Duke University School of Medicine Durham North Carolina; ^2^ Department of Medicine Georgetown University School of Medicine Washington District of Columbia; ^3^ Department of Renal Medicine Salford Royal NHS Foundation Trust Salford UK; ^4^ Department of Medicine, Division of Hematology Oncology UCLA School of Medicine Los Angeles California; ^5^ Department of Clinical and Non‐clinical Research Pharmacosmos A/S Holbaek Denmark; ^6^ Department of Renal Medicine Hull University Teaching Hospitals NHS Trust Kingston upon Hull UK

To the Editor:

Iron deficiency anemia (IDA) is a common problem that causes fatigue and increases risks of morbidity and mortality.^1^ Compared with oral iron, treatment with intravenous (IV) iron may result in better adherence, fewer medical visits, more efficient correction of IDA, and overall improvement in quality of life.

Intravenous iron has been associated with a number of safety concerns, most notably serious hypersensitivity reactions, instilling reluctance within the medical community to use it. High quality clinical trials are warranted to evaluate the safety of IV iron and to compare incidence rates of serious or severe hypersensitivity reactions in response to different IV iron formulations, but small patient numbers in most existing trials have limited the statistical power needed to detect differences in serious or severe hypersensitivity reactions as these are relatively rare.

The FERWON program, which consists of two trials including a total of 3050 patients with IDA due to a broad variety of clinical diagnoses (FERWON‐IDA)^2^ or due to non‐dialysis‐dependent chronic kidney disease (CKD; FERWON‐NEPHRO),^3^ was powered to compare serious or severe hypersensitivity reactions of ferric derisomaltose (FDI), also known as iron isomaltoside 1000, with iron sucrose (IS). As previously reported, the co‐primary safety endpoint was achieved in both trials individually; FDI was associated with a frequency of serious or severe hypersensitivity reactions of 0.3% in both trials. In addition, the incidence of composite cardiovascular adverse events (AEs) was significantly lower in the FDI vs IS group in the FERWON‐NEPHRO trial.^3^ In the efficacy analyses of the individual FERWON‐IDA and FERWON‐NEHRO trials, FDI induced a more rapid hematological response compared to IS and demonstrated non‐inferiority on change in hemoglobin from baseline to week eight.^2^, ^3^

Here, we present the results of the pre‐specified combined safety analysis of the FERWON‐IDA/NEPHRO trials, the aim of which was to evaluate the safety of FDI and IS in a large population of patients with IDA. The primary pre‐specified safety endpoint was the incidence of serious or severe hypersensitivity reactions reported during or after the first dose of randomized treatment. The secondary pre‐specified safety endpoints included the pooled incidence of composite cardiovascular adverse events (AEs) and time to first composite cardiovascular AE. Adjudication of both serious or severe hypersensitivity reactions and composite cardiovascular AEs was performed in a blinded fashion by an independent Clinical Endpoint Adjudication Committee. Hypersensitivity was defined by a standardized set of Medical Dictionary for Regulatory Activities (MedDRA) terms based on discussions with the US Food and Drug Administration (FDA).^2^, ^3^

A total of 5668 patients were screened of whom 3050 were randomized 2:1 to the FDI group (N = 2036) or IS group (N = 1014); 2803 (92%) completed the trial. A total of 2008 patients received a single administration of FDI at a mean ± SD dose of 984 ± 114 (median: 1000) mg, and 1000 received one to five 200 mg administrations (mean: 4.6, median: 5 administrations) of IS at a mean cumulative dose of 902 ± 207 (median: 1000) mg.

A total of 256 potential hypersensitivity reactions in 159 (5.3%) patients were referred to the adjudication committee for blinded assessment. No statistically significant differences were observed between treatment groups in the incidences of mild, moderate, or severe hypersensitivity reactions. Adjudicated serious or severe hypersensitivity reactions were confirmed in six out of 2008 patients (0.3%; 95% confidence interval [CI]: 0.11; 0.65) in the FDI group vs two out of 1000 patients (0.2%; 95% CI: 0.02; 0.72) in the IS group. The risk difference between FDI and IS was estimated to be 0.10% (95% CI: −0.57; 0.48), confirming non‐inferiority of FDI based on the upper limit of the 95% CI for the risk difference being below the non‐inferiority margin of 1.5%‐points.

The incidence of composite cardiovascular AEs was significantly lower in the FDI group compared to the IS group (63 events in 50 [2.5%] patients vs 48 events in 41 [4.1%] patients; *P* = .018). The most frequent cardiovascular AEs in the IS group were hypertension (0.6% in the FDI group vs 1.4% in the IS group, *P =* .062), congestive heart failure (0.3% in the FDI group vs 1.1% in the IS group, *P =* .021), and atrial fibrillation (0.2% in the FDI group vs 0.6% in the IS group, *P =* .093). The time to first composite cardiovascular AE after the first administered dose was significantly longer for FDI vs IS (*P* = .014).

A total of 313 adverse drug reactions (ADRs, ie, related or possibly related adverse events) were reported in 172 (8.6%) patients in the FDI group and 181 ADRs were reported in 90 (9.0%) patients in the IS group (*P* = .68). The most common ADRs (≥1%) were nausea (1.2% in the FDI group and 1.1% in the IS group), rash (1.0% vs 0.1%), dysgeusia (0.2% vs 1.0%), and overdose (0% vs 1.0%). In a post‐hoc analysis of recurrent ADRs in which patients were not censored based on a previously reported ADR (patients were counted one time per day when they experienced ≥1 ADR on a given day), a total of 172 (8.6%) patients experienced ≥1 ADR on 194 distinct days in the FDI group, and 90 (9.0%) experienced ≥1 ADR on 144 distinct days in the IS group. The risk ratio comparing FDI vs IS was 0.67 in favor of FDI (95% CI: 0.56; 0.78, *P* < .001, Figure 1).

**FIGURE 1:**
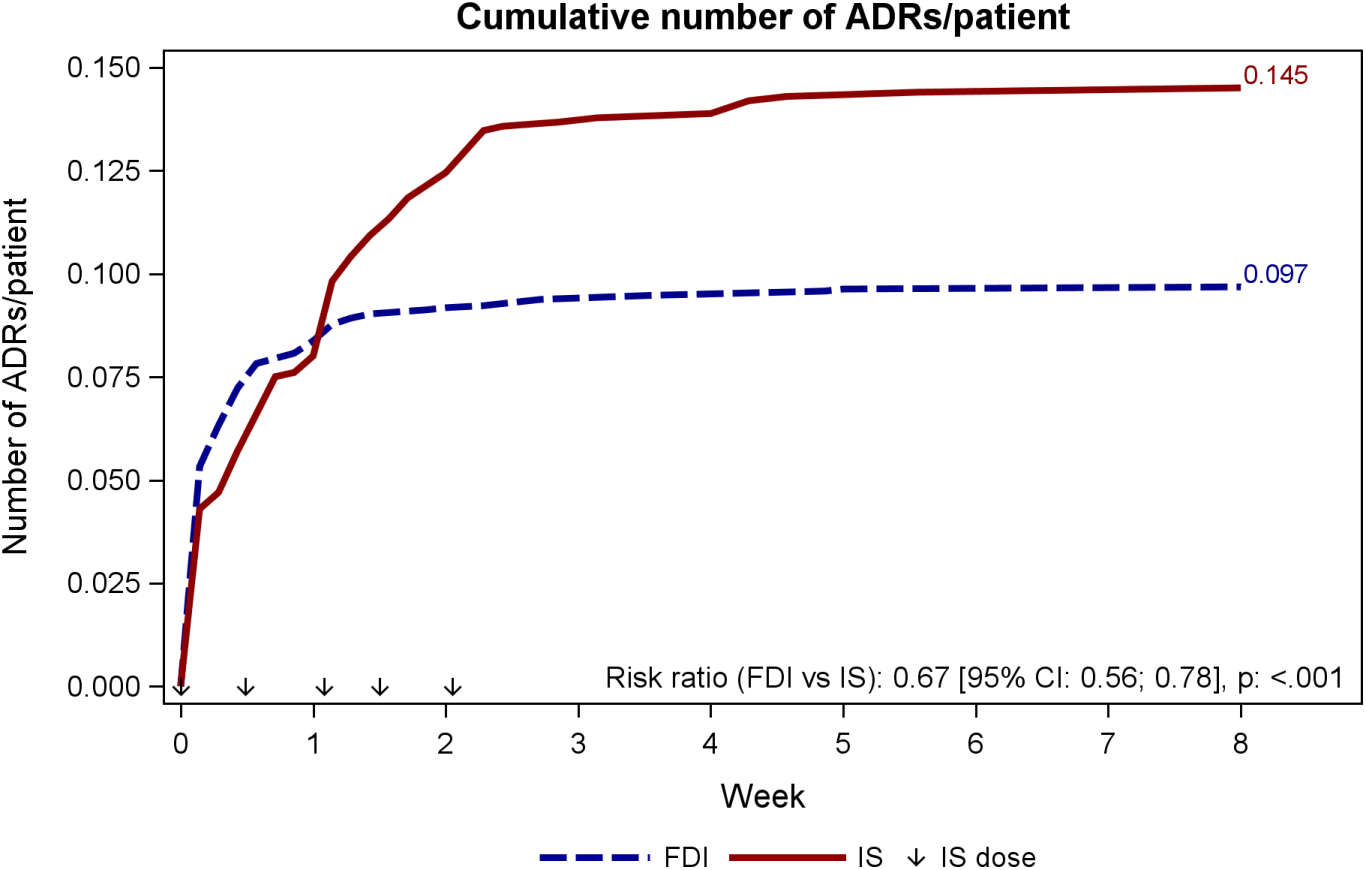
Cumulative number of ADRs/patient. Number of ADRs is modelled by poisson regression with treatment as factor and log values of study duration were used as offset. To account for overdispension, estimates are scaled with the deviance Time since treatment start is calculated as AE start date ‐ treatment start date. Patients are counted once per day. FDI, ferric derisomaltose/iron isomaltoside 1000; IS, iron sucrose

In additional post‐hoc analyses, there were no statistically significant differences between FDI and IS in the incidence of mild, moderate, or severe ADRs. There were four unrelated fatalities in the FDI group (septic shock, cardiac arrest, bile duct cancer, and unknown cause of death,) and three in the IS group (cardiac arrest, exacerbation of congestive heart failure, and drug hypersensitivity to angiotensin‐converting enzyme [ACE] inhibitor).

This pre‐specified combined analysis of the FERWON‐IDA and FERWON‐NEPHRO trials confirms that serious or severe hypersensitivity reactions with IV iron are rare. The PHOSPHARE trials, which are the first published head‐to‐head trials of FDI vs ferric carboxymaltose (FCM), showed a similarly low frequency of serious or severe hypersensitivity reactions with IV iron (0.8% for FDI vs 1.7% for FCM) in a pooled analysis of 245 patients with IDA.^4^

The most extensive and robust analysis to date of serious or severe hypersensitivity reactions with IV iron formulations was published by Pollock and Biggar.^5^ They included safety data from 8599 patients (including the FERWON trials) treated with FDI, FCM, or IS and confirmed that serious or severe hypersensitivity reactions with IV iron administration are rare and that the risk was lower with FDI relative to FCM and IS.^5^

The incidence of composite cardiovascular AEs was significantly lower in the FDI group compared to the IS group. As expected, the FERWON‐NEPHRO trial had a higher overall frequency of composite cardiovascular AEs than the FERWON‐IDA trial given that CKD significantly increases risk of cardiovascular events.^6^ Although there was no statistically significant difference between groups in the number of patients with composite cardiovascular AEs in the FERWON‐IDA trial,^2^ in FERWON‐NEPHRO patients treated with FDI experienced significantly fewer cardiovascular AEs than those treated with IS (4.1% vs 6.9%; *P* = .025).^3^ This suggests that the difference in risk of cardiovascular AEs is more pronounced in a population with a higher risk of cardiovascular complications such as patients with CKD.

There was no difference in the percentage of patients who experienced an ADR in the treatment groups; however, when recurrent ADRs were analyzed, there was a statistically significant difference between the treatment groups in favor of FDI. Thus, patients treated with FDI experienced fewer days with drug related side effects compared to those receiving IS.

In conclusion, both FDI and IS treatments were effective and well tolerated in patients with IDA with or without non‐dialysis‐dependent CKD. The incidence of blindly adjudicated serious or severe hypersensitivity reactions was low for both FDI and IS and non‐inferiority of FDI was demonstrated. The incidence of blindly adjudicated composite cardiovascular AEs was significantly lower with FDI compared to IS. This demonstrates that the more convenient possibility of administrating 1000 mg FDI in one dose rather than up to five doses with IS does not compromise safety and may reduce cardiovascular risks.

## CONFLICT OF INTEREST

Myles Wolf has received consultancy fees from Pharmacosmos A/S, Akebia, Amag, Ardelyx, Bayer, and AstraZeneca.

Michael Auerbach receives research funding for data management from AMAG Pharmaceuticals.

Philip A. Kalra has received personal fees and non‐financial support from Pharmacosmos A/S, grants and personal fees from Vifor Pharma, and grants from Astellas.

John Glaspy has been an advisor to AMAG Pharmaceuticals.

Lars L. Thomsen is employed by Pharmacosmos A/S.

Sunil Bhandari has received honorarium, consultancy fees, membership advisory board, and travel funding from Pharmacosmos A/S, Vifor Pharma, and Astellas.

This work was funded by Pharmacosmos A/S and the investigators/institutions received a fee per patient.

## Data Availability

Individual subject data will not be available; however summarized data may be provided on request.
